# Labour companionship and women’s experiences of mistreatment during childbirth: results from a multi-country community-based survey

**DOI:** 10.1136/bmjgh-2020-003564

**Published:** 2020-11-23

**Authors:** Mamadou Dioulde Balde, Khalidha Nasiri, Hedieh Mehrtash, Anne-Marie Soumah, Meghan A Bohren, Boubacar Alpha Diallo, Theresa Azonima Irinyenikan, Thae Maung Maung, Soe Soe Thwin, Adeniyi K Aderoba, Joshua P Vogel, Nwe Oo Mon, Kwame Adu-Bonsaffoh, Özge Tunçalp

**Affiliations:** 1 Cellulle de Recherche en Sante de la Reproduction en Guinee (CERREGUI), Conakry, Guinea; 2 Schulich School of Medicine and Dentistry, London, Ontario, Canada; 3 Department of Sexual and Reproductive Health and Research, including UNDP/UNFPA/UNICEF/WHO/World Bank Special Programme of Research, Development and Research Training in Human Reproduction (HRP), World Health Organization, Geneve, Switzerland; 4 Centre for Health Equity, University of Melbourne School of Population and Global Health, Melbourne, Victoria, Australia; 5 Obstetrics and Gynaecology, Faculty of Clinical Schools, University of Medical Sciences Teaching Hospital Complex, Akure, Ondo, Nigeria; 6 Department of Medical Research, Yangon, Myanmar; 7 Obstetrics and Gynaecology, Mother and Child Hospital, Akure, Ondo, Nigeria; 8 Maternal, Child, and Adolescent Health Programme, Burnet Institute, Melbourne, Victoria, Australia; 9 Department of Obstetrics and Gynecology, Korle-Bu Teaching Hospital, Accra, Ghana

**Keywords:** maternal health, obstetrics, health policy, health systems, community-based survey

## Abstract

**Background:**

Evidence has shown the benefits of labour companions during childbirth. Few studies have documented the relationship between the absence of labour companions and mistreatment of women during childbirth in low-income and middle-income countries using a standardised tool.

**Methods:**

We conducted a secondary analysis of the WHO multi-country study on how women are treated during childbirth, where a cross-sectional community survey was conducted with women up to 8 weeks after childbirth in Ghana, Guinea, Nigeria and Myanmar. Descriptive analysis and multivariable logistic regression were used to examine whether labour companionship was associated with various types of mistreatment.

**Results:**

Of 2672 women, about half (50.4%) reported the presence of a labour companion. Approximately half (49.6%) of these women reported that the timing of support was during labour and after childbirth and most of the labour companions (47.0%) were their family members. Across Ghana, Guinea and Nigeria, women without a labour companion were more likely to report physical abuse, non-consented medical procedures and poor communication compared with women with a labour companion. However, there were country-level variations. In Guinea, the absence of labour companionship was associated with any physical abuse, verbal abuse, or stigma or discrimination (adjusted OR (AOR) 3.6, 1.9–6.9) and non-consented vaginal examinations (AOR 3.2, 1.6–6.4). In Ghana, it was associated with non-consented vaginal examinations (AOR 2.3, 1.7–3.1) and poor communication (AOR 2.0, 1.3–3.2). In Nigeria, it was associated with longer wait times (AOR 0.6, 0.3–0.9).

**Conclusion:**

Labour companionship is associated with lower levels of some forms of mistreatment that women experience during childbirth, depending on the setting. Further work is needed to ascertain how best to implement context-specific labour companionship to ensure benefits while maintaining women’s choices and autonomy.

**Key questions**

**What is already known?**

- Two Cochrane reviews have shown that the support provided by a labour companion during labour and childbirth improves maternal and perinatal outcomes, including enhancing the physiological process of labour and helping women have positive childbirth experiences.
- These available reviews have also demonstrated that women greatly value and benefit from the presence of a support person of choice during labour and childbirth who can provide emotional, psychological and practical support and advice to women during labour and childbirth.
- There is limited and varied evidence using empirical data to examine the association between labour companionship and mistreatment of women during childbirth.

**What are the new findings?**

- We provide evidence that women without labour companions experienced some, but not all, forms of mistreatment more often than women with labour companions, and that this association varied depending on the country.
- Using a standardised tool to examine women’s mistreatment during childbirth in four low/middle-income countries facilitates comparability of the results across settings and contexts.

**Key questions**

**What do the new findings imply?**

- Our study shows that labour companions can be important in promoting respectful maternity care, however the associations vary by context.
- Health careHealthcare facilities can establish supportive policies that allow and encourage women to have companions during labour and childbirth.
- Furthermore, allowing women to have the support of a companion of choice during labour and childbirth is a practical intervention that can be implemented to improve provision and experience of care.

## Introduction

Women have a right to a positive experience of care during pregnancy and childbirth. According to the WHO’s Quality of Care Framework for maternal and newborn health, a core component of quality care is the experience of care, which includes effective communication, respectful and dignified care, and access to emotional and social support of the women’s choice.^1^ Despite the importance of women’s experiences of care, evidence from a WHO multi-country study showed that more than one-third of women experienced mistreatment during childbirth in health facilities across four countries.^2^ Mistreatment during childbirth can amount to implications for human rights and the disempowerment of women, and negatively impact future care-seeking behaviours.^3–6^ Emotional and social support can be provided to women by a companion of choice during labour and childbirth (hereafter referred to as labour companions), which refers to support provided to women during labour and childbirth by a person of her choice, such as a husband or partner, family member or friend.^7^ A 2017 Cochrane effectiveness review identified important benefits of labour companionship for the woman and her baby, including increased spontaneous vaginal birth, shorter duration of labour, better Apgar scores, reductions in caesarean birth, use of analgesia and a reduction in negative birth experiences.^7^ Labour companionship has also been linked to increased satisfaction with maternity services.^8 9^ The 2018 WHO recommendations on intrapartum care for a positive childbirth experience recommend a labour companion of choice for all women giving birth.^10^ Access to family and community support (including the presence of a labour companion) has also been identified as a core component of respectful maternity care.^11^

A 2019 Cochrane qualitative evidence synthesis reported that labour companions support women by acting as advocates, bridging communication gaps between health workers and women, and providing practical and emotional support (such as massages, encouraging mobility, praise and reassurance).^12^ In the past decade, quantitative studies^13–21^ have explored general perceptions and factors associated with labour companionship in maternity care (antenatal care, labour and childbirth and/or postnatal care), but to date have not explored the relationship between labour companionship and mistreatment. Furthermore, despite not always being integrated within studies that assess experience of care,^13 16 17 20^ the perspective of the woman is imperative to design maternity care that responds to women’s needs. A critical research gap is therefore using standardised tools to quantitatively measure the association between the presence of a labour companion and the experience of mistreatment during childbirth.

This study used data from the WHO multi-country study ‘How women are treated during facility-based childbirth’ in Ghana, Guinea, Nigeria and Myanmar,^22 23^ to describe the characteristics of labour companionship in maternity care settings and explore the relationship between labour companionship and the different types of mistreatment during childbirth.

## Methods

### Study design and participation

This study is a secondary analysis of data that was collected for a larger cross-sectional study designed to develop and validate two tools to measure the mistreatment of women during childbirth in health facilities in Ghana, Guinea, Nigeria and Myanmar. The protocol for the formative phase and methodological development of these tools is available^22 23^ and the methods and results of the primary analysis have been published.^2^ Briefly, in each country, three facilities were purposively selected based on the following inclusion criteria^22^ : (1) facilities not included in the formative phase of developing these tools; (2) secondary-level facility or higher; (3) ≥200 births per month and (4) well-defined community catchment area. This analysis uses the community-based survey, which was conducted with women up to 8 weeks post partum.

### Data collection

#### Participants

Pregnant women in established labour (as per clinical assessment), who were admitted to participating facilities for childbirth during the study period were approached by data collectors to participate in the study. Women were eligible to participate if they were 15 years of age or older, willing and able to participate, and available for a follow-up interview up to 8 weeks post partum. Women were ineligible to participate, if they were admitted for reasons other than childbirth; were a first-degree relative of a facility employee; were distressed or otherwise unable to reasonably provide consent; resided outside of the facility catchment area (defined for each facility) or were unable to provide enough contact information. The women were contacted at 2–3 weeks post partum to schedule a follow-up interview at a time and private place of their convenience. During the follow-up interview, the data collectors reconfirmed the woman’s consent to participate in the study and administered the community survey on a digital, tablet-based tool (BLU Studio XL2, Android, BLU Products, Miami, Florida, USA). Women who could not be reached for follow-up after three attempts were recorded as lost to follow-up. Recruitment continued until the target facility sample size was reached.^23^ Interviews were administered in a local or national language (English, French, Twi, Yoruba, Sousou, Malinke, Poular and Burmese).

#### Measurement tool

The community survey tool was developed using an iterative mixed-methods approach which is described in detail elsewhere.^22 23^ In short, the survey domains and questions are organised according to the typology of mistreatment of women during childbirth developed from a systematic review.^2^ The survey was interviewer-administered and comprised of two forms: a screening form (completed at recruitment) and a survey form (completed during the survey). The survey form had three main sections: general information (sociodemographic information and obstetric history), mistreatment during care (physical abuse, verbal abuse, stigma or discrimination, vaginal examination, pain relief, neglect and labour companionship) and outcomes (eg, childbirth outcomes, maternal interventions, satisfaction with care).

In this analysis, the independent variable was the presence or absence of a labour companion at any point during care at the facility, as reported by women. The outcomes of interest were dichotomous variables indicating the presence (yes/no) of any physical abuse (beating, slapping, kicking, pinching or physically restraining women), any verbal abuse (insulting, threatening or blaming women), any stigma or discrimination (discrimination based on sociodemographic or medical characteristics), lack of informed consent during procedures (procedure not explained or no permission given), ineffective/poor communication (concerns were not listened or responded to), neglect and abandonment (three subcategories: provider absent at time of birth, long wait times to be seen by a health worker, and feeling ignored, neglected or like a nuisance) and pain relief (requested and not received). We also included a composite variable which combined the presence of any one or more of physical abuse, verbal abuse, or stigma or discrimination.

### Data analysis

Descriptive analysis was performed to explore both pooled and country-stratified labour companion characteristics including timing of support and identity of the labour companion. Myanmar was excluded from further analyses because 99.7% (629/631) of women reported the presence of a labour companion. Women reporting labour companionship status as unknown in any country (0.3%; 8/2672) were also excluded from further analyses. Descriptive analyses and Χ^2^ tests were conducted to assess sociodemographic characteristics, obstetric characteristics and mistreatment typology by presence or absence of a labour companion (also presented by country as online supplemental tables). Multivariable logistic regression models were fitted to evaluate the association between the presence of a labour companion and the mistreatment variables of interest. For this analysis, our assumption was that all women would have the opportunity to have a labour companion at some point during labour, childbirth and/or after childbirth regardless of mode of birth. Due to the presence of effect modification by country, models were stratified by country. All models were adjusted for maternal age, education, marital status and parity. Data were analysed using SAS V.9.4.

10.1136/bmjgh-2020-003564.supp1 Supplementary data

### Patient and public involvement

A technical consultation with representatives from advocacy groups, non-governmental organisations, research organisations, universities, professional associations and United Nations agencies was held at the WHO in November 2013 and informed the design of this study. Women who recently gave birth were involved in content validity testing and providing feedback on the validity testing of the community survey tool.^23^

## Results

Table 1 shows the pooled and country-stratified characteristics of labour companions. Across the four countries (N=2672), 50.4% (1346/2672) women reported a labour companion at any point during care at the facility, though this varied between countries—395/2672 (47.3%) in Ghana, 82/2672 (12.7%) in Guinea, 629/2672 (23.5%) in Myanmar and 240/2672 (42.8%) in Nigeria. Among women reporting the presence of a labour companion at any point during care (n=1346), 18.7% of women had one or more labour companions present across all timepoints of support (labour, childbirth and after childbirth), ranging from 2.2% in Guinea to 29.2% in Nigeria. Labour companions were present most often only after childbirth in Ghana (138/395; 34.9%) and Guinea (36/82; 43.9%), and during both labour and after childbirth (but not during childbirth) in Myanmar (432/629; 68.7%) and Nigeria (103/240; 42.9%). The most common person acting as a labour companion both overall and by country were family members only (632/1346; 47.0%), except Nigeria where it was male partners (115/240; 47.9%). After excluding Myanmar (n=630) and unknowns (n=8), 2034 women were included in the remainder of the analyses.

**Table 1:** Characteristics of labour companionship by country (N=2672)*

	Ghana	Guinea	Myanmar	Nigeria	Total
	n (%)	n (%)	n (%)	n (%)	n (%)
Total number of women with labour companion present at any point during care at the facility	395 (47.3)	82 (12.7)	629 (99.7)	240 (42.8)	1346 (50.4)
Total number of women with labour companion absent at any point during care at the facility	437 (52.3)	560 (86.9)	1 (0.16)	320 (57.0)	1318 (49.3)
Unknown	4 (0.5)	2 (0.2)	1 (0.2)	1 (0.2)	8 (0.3)
Timing of support (mutually exclusive categories) (N=1346)					
Labour only	45 (11.4)	9 (10.9)	2 (0.32)	15 (6.3)	71 (5.3)
Childbirth only	5 (1.3)	1 (1.2)	1 (0.16)	4 (1.7)	11 (0.8)
After childbirth only	138 (34.9)	36 (43.9)	2 (0.3)	18 (7.5)	194 (14.4)
During labour and childbirth	27 (6.8)	1 (1.2)	46 (7.3)	22 (9.2)	96 (7.1)
Labour and after childbirth	105 (26.6)	27 (32.9)	432 (68.7)	103 (42.9)	667 (49.6)
During childbirth and after childbirth	20 (5.1)	6 (7.3)	19 (3.0)	8 (3.3)	53 (3.9)
Labour, childbirth and after childbirth	54 (13.7)	2 (2.2)	126 (20.0)	70 (29.2)	252 (18.7)
Unknown	1 (0.3)	0	1 (0.16)	0	2 (0.15)
Labour companion (mutually exclusive categories) (N=1346)					
Male partner only	151 (38.2)	4 (4.9)	41 (6.5)	115 (47.9)	311 (23.1)
Family member only	151 (38.2)	72 (87.8)	338 (53.7)	71 (29.6)	632 (47.0)
Both (male partner and family member)	76 (19.2)	3 (3.7)	203 (32.3)	35 (14.6)	317 (23.6)
Other (friend(s)), doula(s), traditional birth attendants)	17 (4.3)	3 (3.7)	47 (7.5)	19 (7.9)	86 (6.4)

*Overall sample: N=2672. Ghana: 836 (31.3%), Guinea: 644 (24.1%), Myanmar: 631 (23.6%), Nigeria: 561 (21.0%).

Table 2 shows the sociodemographic and obstetric characteristics of women by presence or absence of a labour companion. Women without a labour companion were significantly more likely to be younger, married, have no education, have ≥2 prior births, and initiate breastfeeding between 1 and 24 hours of birth. Online supplemental table 1 shows that there were country-level differences in maternal age, marital status, education, parity, gravidity and breastfeeding initiation among women with an absence of labour companion (n=1317).

**Table 2:** Sociodemographic information and obstetric characteristics of women by labour companion status, N=2034

	No labour companion present (N=1317)	Labour companion present (N=717)	Total* (N=2034)
	n (%)	n (%)	n (%)
Maternal age†			
15–19	195 (14.8)	51 (7.1)	246 (12.1)
20–29	649 (49.4)	327 (45.8)	976 (48.1)
≥30	471 (35.8)	336 (47.1)	807 (39.8)
Unknown	2 (0.1)	3 (0.4)	5 (0.2)
Marital status†			
Currently married/cohabiting	1191 (90.6)	625 (87.7)	1816 (89.6)
Single‡	123 (9.4)	88 (12.3)	211 (10.4)
Other/do not know/unknown/missing	3 (0.2)	4 (0.6)	7 (0.3)
Education†			
No education	273 (20.8)	50 (7.0)	323 (15.9)
Some primary	125 (9.5)	55 (7.7)	180 (8.9)
Complete primary	374 (28.4)	180 (25.2)	554 (27.3)
Complete secondary	310 (23.6)	226 (31.7)	536 (26.4)
Complete tertiary	213 (16.2)	180 (25.2)	393 (19.4)
Vocational/other/unknown	22 (1.7)	26 (3.6)	48 (2.4)
Number of previous births†			
1	747 (56.8)	453 (63.5)	1200 (59.1)
2 or 3	377 (28.6)	183 (25.5)	560 (27.5)
4+	191 (14.5)	78 (10.9)	269 (13.2)
Unknown	2 (0.2)	3 (0.4)	5 (0.2)
Number of previous pregnancies			
1	397 (30.2)	194 (27.2)	591 (29.1)
2 or 3	535 (40.6)	311 (43.4)	846 (41.6)
4+	381 (29.0)	209 (29.3)	590 (29.1)
Unknown	3 (0.2)	3 (0.4)	6 (0.3)
Number of babies at most recent birth			
1 (singleton)	1285 (97.7)	699 (97.9)	1984 (97.8)
2 (twins)	30 (2.3)	15 (2.1)	45 (2.2)
Unknown	2 (0.2)	3 (0.4)	5 (0.2)
Breast feeding			
Currently breast feeding†	1258 (95.5)	698 (97.4)	1956 (96.2)
Breastfeeding initiation† (from time of most recent birth) (N=1971)			
<1 hour	552 (43.5)	338 (48.2)	890 (45.2)
≥1–<24 hours	611 (48.2)	273 (38.9)	884 (44.9)
≥24 hours	104 (8.2)	91 (13.0)	195 (9.9)
Unknown/missing	2 (0.2)	0	2 (0.1)

*Total is sample based on Ghana, Guinea and Nigeria.

†Significant Χ^2^ at p<0.05.

‡Single, separated, divorced or widowed.

Table 3 shows the distribution of mistreatment variables by presence or absence of a labour companion and online supplemental table 2 shows country-disaggregated distributions. Women without a labour companion were significantly more likely to report physical abuse (189/1317; 14.4% vs 76/717; 10.6%). No significant differences were observed between women with and without a labour companion for reporting any form of verbal abuse, or stigma and discrimination. Among women without a labour companion, physical abuse ranged from 4.4% (19/437) in Ghana to 21.3% (119/560) in Guinea (online supplemental table 2). Furthermore, among women without a labour companion, women in Nigeria were more likely to report any form of physical or verbal abuse, or stigma and discrimination (160/320; 50.0%) compared with Guinea (221/560; 39.5%) and Ghana (152/437; 34.8%).

**Table 3:** Mistreatment among women with and without a labour companion present at any point during care, N=20 34*

	No labour companion present (N=1317)	Labour companion present (N=717)	Total† (N=2034)
	n (%)	n (%)	n (%)
Any physical abuse, verbal abuse, or stigma and discrimination	533 (40.5)	280 (39.1)	813 (40.0)
Any physical abuse‡	189 (14.4)	76 (10.6)	265 (13.0)
Any verbal abuse	447 (33.9)	257 (35.8)	704 (34.6)
Any stigma and discrimination	40 (3.0)	28 (3.9)	68 (3.3)
Informed consent for procedures			
C-section‡	121 (9.2)	91 (12.7)	212 (10.4)
Non-consented*	20 (18.5)	10 (12.4)	30 (14.2)
Episiotomy*‡¶	177 (14.8)	103 (19.0)	276 (16.1)
Non-consented*§	87 (56.8)	42 (44.2)	129 (52.0)
Induction of labour‡	68 (5.6)	105 (17.4)	173 (9.5)
Non-consented*	14 (21.9)	20 (21.5)	34 (21.7)
Any vaginal examination‡	1276 (96.9)	659 (91.9)	1935 (95.1)
Non-consented‡*	701 (55.6)	278 (43.1)	927 (51.4)
Communication			
Woman felt that health workers or staff did not listen and respond to her concerns‡	212 (16.3)	65 (9.3)	277 (13.9)
Neglect and abandonment			
Staff member not present when the baby came out§	25 (2.1)	16 (2.6)	41 (2.3)
Woman waited for long periods of time before attended by health workers‡	176 (13.4)	126 (17.6)	302 (14.9)
Woman felt ignored, neglected, or that presence was a nuisance for health workers or staff	191 (14.5)	121 (17.0)	312 (15.4)
Pain relief			
Requested pain relief‡	278 (21.1)	92 (12.9)	370 (18.2)
Did not receive pain relief	103 (37.1)	33 (35.9)	136 (36.8)

*Percentages exclude unknowns for each variable.

†Total is sample based on Ghana, Guinea and Nigeria.

‡Significant Χ^2^ at p<0.05.

§Marginally significant (p=0.05).

¶Among women with vaginal birth only.

In terms of informed consent for procedures, women without a labour companion were more likely to receive non-consented care for vaginal examinations (701/1317; 55.6%) compared with women with a labour companion (278/717; 43.1%). There was no significant difference in the prevalence of consent for caesarean sections, episiotomies and induction of labour in pooled analyses (table 3); however, there were significant country-level variations in non-consented procedures (caesarean sections and episiotomies) (online supplemental table 2). For example, women without a labour companion were most likely to have non-consented caesarean sections in Ghana (15/52; 28.9%) and non-consented episiotomies in Guinea (35/46; 77.8%). In our sample, women with a labour companion were significantly more likely to have undergone episiotomy (103/717; 19.0%), induction of labour (105/717; 17.4%) or a caesarean section (91/717; 12.7%), compared with women without a labour companion (173/1317, 14.8%; 68/1317; 5.6% and 121/1317; 9.2%, respectively).

In terms of communication, women without a labour companion were more likely to report that health staff did not listen or respond to their concerns (212/1317; 16.3%) compared with women with a labour companion (65/717; 9.3%). Among women without a labour companion, this ranged from 12.8% (41/320) in Nigeria to approximately one in six women (71/437; 16.3%) in Ghana and 17.9% (100/560) in Guinea (online supplemental table 2).

In addition, women with a companion were significantly more likely to report waiting for long periods of time before being attended by health workers (126/717; 17.6%) compared with women without a companion (176/1317; 13.4%), with the highest prevalence in Ghana (178/437; 40.7%) (table 3 and online supplemental table 2). In pooled analyses, there was no significant difference in women feeling ignored, neglected or that their presence was a nuisance for health personnel (121/717; 17.0% with a companion vs 191/1317; 14.5% without). Women with a labour companion were less likely to request pain relief (92/717; 12.9% vs 278/1317; 21.2%). There was no significant difference in whether pain relief was received once requested between women with and without a companion in the pooled analysis. At the country-level, there was significant variation: 65.6% (120/183) of women in Guinea requested but did not receive pain relief, compared with 31.2% (19/61) in Ghana and 41.2% (14/34) in Nigeria.

Table 4 shows the results of the adjusted multivariable logistic regression model which examined whether the absence of a labour companion is associated with a woman’s experience of mistreatment during childbirth, adjusting for maternal age, education, parity and marital status. Overall, the relationship between the absence of a labour companion and types of mistreatment varied by country. The strongest associations between the absence of a labour companion and experiences of mistreatment was in Guinea, where women without a labour companion were more likely to report any form of mistreatment (physical abuse or verbal abuse, or stigma and discrimination) compared with women who did have a labour companion (adjusted OR/AOR: 3.6, 95% CI 1.9–6.9). In Guinea, the absence of labour companionship was significantly associated with a higher likelihood of reporting physical abuse (AOR: 5.2, 95% CI 1.8–14.4) whereas in Ghana it was associated with a lower likelihood (0.5, 95% CI 0.3–0.9; Nigeria: not significant (NS)). In Guinea, there was also a significant association between the absence of labour companionship and verbal abuse (AOR: 2.7, 95% CI 1.3–5.5; Ghana: NS; Nigeria: NS). Non-consented vaginal examinations were more than two times as likely to be conducted among women without a labour companion in Guinea and Ghana (Guinea: AOR: 3.2, 95% CI 1.6–6.4; Ghana: AOR: 2.3, 95% CI 1.7–3.1; Nigeria: NS). Poor communication was more likely to be reported by women without a labour companion in Ghana (AOR: 2.0, 95% CI 1.3–3.2; Nigeria: NS; Guinea: NS). Women without a labour companion in Nigeria were less likely to report waiting for long periods of time (AOR: 0.6, 95% CI 0.3–0.9; Ghana: NS; Guinea: NS).

**Table 4:** Association between absence of a labour companion and mistreatment*†‡

	Any physical abuse, verbal abuse, or stigma or discrimination	Physical abuse	Verbal abuse	Non-consented vaginal examination	Poor communication between healthcare providers and women	Felt ignored, neglected or their presence was a nuisance to health workers	Waited for long periods of time to be attended by health workers
	AOR (95% CI)	AOR (95% CI)	AOR (95% CI)	AOR (95% CI)	AOR (95% CI)	AOR (95% CI)	AOR (95% CI)
Ghana	0.8 (0.6–1.06)	**0.5** (**0.3–0.9**)	0.8 (0.6–1.1)	**2.3** (**1.7–3.1**)	**2.0** (**1.3–3.2**)	0.7 (0.5–1.1)	1.0 (0.7–1.4)
Guinea	**3.6** (**1.9–6.9**)	**5.2** (**1.8–14.4**)	**2.7** (**1.3–5.5**)	**3.2** (**1.6–6.4**)	1.3 (0.6–2.5)	0.8 (0.4–1.7)	1.0 (0.5–2.3)
Nigeria	1.2 (0.8–1.6)	1.0 (0.6–1.5)	1.1 (0.8–1.6)	1.2 (0.8–1.7)	1.8 (0.9–3.2)	1.3 (0.8–2.0)	**0.6** (**0.3–0.9**)

Bold=significant Χ^2^ at p<0.05.

*AORs adjusted for maternal age, education, marital status and parity.

†Reference is labour companion present at any point during care.

‡Myanmar is not included in the model because 99.7% of women reported having a labour companion.

AOR, adjusted OR.

## Discussion

We report the results of a community-based survey of postpartum women in Nigeria, Ghana, Guinea and Myanmar where we described characteristics of labour companionship and explored the association between labour companionship and experiences of mistreatment. Half (50.4%) of the women in our sample reported the presence of a companion at any point during facility-based care. These results are aligned with a 2018 WHO policy survey among Ministries of Health, where the presence of a companion of choice during labour and childbirth varied globally (Africa (52%), Americas (75%), Eastern Mediterranean (19%), South East Asian region (72%), Western Pacific (42%)).^24^ There is not a standardised approach used to ascertain the presence, duration and timing of labour companionship which presents challenges for facilitating comparability between studies.^25^ For example, Afulani *et al*^26^ used both a Likert scale and binary yes/no question to ascertain the presence or absence of labour companionship in Kenya among women up to 9 weeks post partum (78.0%, 26.0% and 88.0% during labour, birth and after birth, respectively), Udofia and Akwaowo^27^ designed semistructured questionnaire for use among women up to 8 weeks post partum (69.4% during childbirth in Uyo, Nigeria) and Craymah *et al*^20^ used a self-designed survey for women up to 12 weeks post partum and only assessed male labour companionship in Anomabo, Ghana (44.0% during birth). Due to the use of a single, standardised tool across four countries, we were able to establish how labour companionship characteristics varied across countries in our study.

In our study, the most common labour companions were family members (47.0%), male partners only (23.1%) or both (23.6%), which is similar to other studies conducted in the Middle East and Africa.^28–34^ Women’s preferences for the type of companion can depend on several factors such as having preference for another relative, feeling embarrassed, or a loss of sexual attraction if supported by a male partner or husband, and negative health staff attitude towards labour companions.^13 14 21 32^ Importantly, gender roles and expectations can be critical influencers of women’s and men’s perception of and participation in labour companionship, as pregnancy and childbirth may be considered as exclusively as ‘women’s business’.^35 36^

In the adjusted model, we found that the association between experiences of mistreatment and absence of a labour companion significantly varied by country. Notably, the absence of a labour companion was associated with more than fivefold increased risk of physical abuse in Guinea, suggesting that support from a labour companion may be protective against mistreatment during childbirth. The absence of a labour companion was also associated with non-consented vaginal examination, which is aligned with other research suggesting that women valued labour companions because they facilitated their involvement in decision-making and informed consent for medical procedures.^36^ Furthermore, the absence of a labour companion was associated with poor communication in Ghana and Nigeria, supporting the evidence that labour companions can act as advocates who improve the communication of women’s preferences to health workers.^7 12 36 37^ Conversely, in Ghana women without a labour companion were less likely to report physical abuse and in Nigeria less likely to report long waiting times, and further research is warranted to better interpret these associations.

Our findings suggest that the relationship between labour companionship and mistreatment is not homogenous, but context-dependent. This highlights the importance of participatory adaptation of labour companionship models across settings to ensure that it meets the needs of key stakeholders including women, their families and health workers. For example, an implementation research study conducted in Syria, Egypt and Lebanon used participatory approaches to develop and implement a tailored labour companionship model and reported improvements in acceptability of labour companions among health workers and women’s satisfaction with care during childbirth.^9^ There are important components of labour companionship implementation that need to be taken into consideration. Creating physical spaces in health facilities or structuring labour wards that support companionship may also be important, such as ensuring the availability and use of privacy measures (curtains/dividers) and a chair for the companion.^12^ For example, according to labour observation data from the health facilities across the four countries, the prevalence of curtains, partitions or other measures used to provide privacy for women ranged between 16.3% in Nigeria and 92.1% in Ghana.^2^ Promoting the benefits of labour companionship and social support for women during labour and childbirth throughout health worker pre-service and in-service training may help to reverse negative attitudes towards labour companions and improve communication between healthcare providers, women and their companions.^12^ Participatory training and supervision could include training for providers on how to integrate companions into care teams, providing information and training for companions during antenatal care, and specifying clear roles and expectations for the companion.^9 12^

In line with previous evidence, our study shows that women with labour companions were less likely to request pain relief.^7^ However, medical procedures (caesarean sections, episiotomies and inductions of labour) were more common when labour companions were present in our study, which is contrary to existing evidence coming predominantly from high-income countries.^7^ A plausible explanation might be that the presence of a labour companion in our study contexts may lead to greater attention from the healthcare provider that in turn may contribute to increased use of medical procedures, whether or not they are clinically indicated. This finding may also be attributable to the differences in the sociodemographic and obstetric characteristics of women in the study presenting with or without labour companions across the health facilities. Exploration of these issues through qualitative studies will be important. Previous research has showed differential care patterns in health facilities based on women’s characteristics, for example, more educated women were more likely to be allowed continuous labour companion support.^26^ It is important to note that the sample sizes for procedures and informed consent for procedures are relatively small therefore the results of these analyses should be interpreted with caution.

### Strengths and limitations

Strengths and limitations of our tool have been previously described in detail elsewhere.^2^ Strengths of our study include the ability to quantitatively explore the association between women’s reported presence of labour companion and mistreatment during childbirth in Nigeria, Ghana, Guinea and Myanmar using an evidence-informed and standardised tool.

Limitations include the possibility that labour companionship may not have been uniformly implemented across the settings in this study. Additionally, we were not able to ascertain how ‘continuous’ the support was for individual women (eg, duration of companionship). For example, some facilities might have not allowed labour companionship at a certain timepoint during care, and these rules about companionship may be differentially applied to women across study settings. To address the issue about the timing of support, we presented descriptive results across time periods during which a companion was reported to be present: during labour, during childbirth and during the postpartum period (and any combination of these periods). Third, by excluding women who were unable to provide their contact information, our study may have excluded women who were more prone to mistreatment. Finally, as the surveys were conducted up to 8 weeks post partum, recall bias is a possibility since a woman’s recollection of her childbirth may be influenced by factors such as her child’s current health status.

### Implications for research and practice

Our study showed that the absence of a labour companion was associated with increased risk of different types of mistreatment. Standardised measurement of labour companionship is needed across different contexts to better understand how continuous support is being implemented. We also need to better understand how such support can be provided in terms of timing of support, acceptability by health providers, feasibility within health systems, types of support (partner, family and/or other companions of choice) and the roles labour companions can play during this critical period. Further research understanding the relationship dynamics between women, companions and providers and on effective and sustainable implementation and scale up of labour companionship in different contexts is needed to ensure that it improves the quality of care and outcomes for women.

## Conclusion

More than half of postpartum women surveyed in Nigeria, Ghana, Guinea and Myanmar reported the presence of a labour companion. Depending on the country, the presence of a labour companion was associated with a lower risk of physical abuse, unconsented vaginal examinations and poor communication with healthcare providers. Allowing women to have a companion of choice can be a low-cost and effective intervention for reducing mistreatment of women during labour and childbirth in low-resource settings. Further research is needed to explore how best to implement labour companionship across different settings and ensure that women’s choices and autonomy are respected.
