# Supplementary material for: Labour companionship and women’s experiences of mistreatment during childbirth: results from a multi-country community-based survey
Source: BMJ Glob Health. 2020 Nov 23;5(Suppl 2):e003564. doi: 10.1136/bmjgh-2020-003564 (PMC7684665; doi:10.1136/bmjgh-2020-003564)
Supplement: Supplementary data [file bmjgh-2020-003564supp001.pdf]

SUPPLEMENTARY FILE

Supplementary Table 1. Sociodemographic information and obstetric history of women with a labour companion present at any point during care by country, $N = 1346$

	**Ghana (N=395)**	**Guinea (N=82)**	**Myanmar (N=629)**	**Nigeria (N=240)**	**Total (N=1346)**
	n (%)	n (%)	n (%)	n (%)	n (%)
Maternal age					
15-19	23 (5.8)	22 (26.8)	38 (6.0)	6 (2.5)	89 (6.6)
20-29	183 (46.3)	44 (53.7)	343 (54.5)	102 (42.5)	672 (49.9)
30+	189 (47.9)	16 (19.5)	248 (39.4)	132 (55.0)	585 (43.5)
Marital status					
Currently married or cohabiting	332 (84.1)	75 (91.5)	611 (97.1)	221 (92.1)	1239 (92.1)
Single[1]	62 (15.7)	7 (8.5)	18 (2.9)	19 (7.9)	106 (7.9)
Other/Do Not Know/Unknown/Missing	1 (0.3)	0 (0.0)	0 (0.0)	0 (0.0)	1 (0.07)
Education					
No education	20 (5.1)	29 (35.4)	15 (2.4)	1 (0.4)	65 (4.83)
Some primary	33 (8.4)	21 (25.6)	104 (16.5)	3 (1.3)	161 (12.0)
Primary	144 (36.5)	17 (20.7)	183 (29.1)	19 (7.9)	363 (27.0)
Secondary	125 (31.7)	6 (7.3)	192 (30.5)	96 (40.0)	419 (31.1)
Post-secondary/tertiary	56 (14.2)	5 (6.1)	135 (21.5)	119 (49.6)	315 (23.4)
Vocational/Other/Unknown	17 (4.3)	4 (4.9)	0 (0.0)	2 (0.8)	23 (1.7)
Number of previous births					
1	277 (70.1)	73 (89.0)	353 (56.1)	103 (42.9)	806 (59.9)
2 or 3	80 (20.3)	5 (6.1)	228 (36.2)	98 (40.8)	411 (30.5)
4+	35 (8.9)	4 (4.9)	47 (7.5)	39 (16.3)	125 (9.3)
Unknown	3 (0.8)	0 (0.0)	1 (0.2)	0 (0.0)	4 (0.3)
Number of previous pregnancies					
1	98 (24.8)	27 (32.9)	323 (51.4)	69 (28.8)	517 (38.4)
2 or 3	170 (43.0)	29 (35.4)	245 (39.0)	112 (46.7)	556 (41.3)
4+	124 (31.4)	26 (31.7)	60 (9.6)	59 (24.6)	269 (20.0)
Unknown	3 (0.8)	0 (0.0)	0 (0.0)	0 (0.0)	3 (0.2)
Singleton pregnancy					
Yes	387 (98.0)	80 (97.6)	619 (98.4)	235 (97.9)	1321 (98.1)
No (Twins/Multiple Birth)	8 (2.0)	2 (2.4)	10 (1.6)	5 (2.1)	25 (1.9)
Currently breastfeeding					
Yes	385 (97.5)	79 (96.3)	614 (97.6)	237 (98.8)	1315 (97.7)
Breast feeding initiation					
<1 hour	192 (49.6)	50 (63.3)	442 (71.4)	99 (41.4)	783 (59.1)
>1 hour but <24 hours	144 (37.2)	26 (32.9)	121 (19.6)	103 (43.1)	394 (29.8)
>24 hours	51 (13.2)	3 (3.8)	56 (9.1)	37 (15.5)	147 (11.1)

[1]Single, separated, divorced or widowed

Supplementary Table 2. Mistreatment among women with absence of a labour companion during care by country, *N*=1317[1]

	**Ghana (N=437)**	**Guinea (N=560)**	**Nigeria (N=320)**	**Total (N=1317)**
	n (%)	n (%)	n (%)	n (%)
Any physical abuse, verbal abuse, or stigma and discrimination*	152 (34.8)	221 (39.5)	160 (50.0)	532 (40.4)
Any physical abuse*	19 (4.4)	119 (21.3)	51 (15.9)	189 (14.3)
Any verbal abuse*	141 (32.3)	161 (28.8)	145 (45.3)	447 (33.9)
Any stigma or discrimination*	15 (3.4)	8 (1.4)	17 (5.3)	40 (3.0)
Informed consent for procedures				
C-section*	52 (11.9)	58 (10.4)	11 (3.4)	121 (9.2)
Non-consented*	15 (28.9)	4 (6.9)	1 (9.1)	20 (16.5)
Missing	9 (17.3)	1 (1.7)	3 (27.3)	13 (10.7)
Episiotomy[2]*	48 (11.0)	46 (8.2)	83 (25.9)	177 (13.4)
Non-consented*	19 (42.2)	35 (77.8)	33 (39.8)	87 (50.3)
Missing	9 (20.0)	2 (4.4)	9 (10.8)	20 (11.5)
Induction of labour*	40 (9.2)	2 (0.4)	26 (8.1)	68 (5.2)
Non-consented	11 (27.5)	1 (50.0)	2 (7.7)	14 (20.6)
Missing	3 (7.5)	0 (0.0)	1 (3.9)	4 (5.8)
Vaginal examination*	427 (97.7)	537 (95.9)	312 (97.5)	1276 (96.8)
Non-consented *	239 (55.9)	278 (51.8)	184 (58.9)	701 (54.9)
Missing	10 (2.3)	2 (0.4)	3 (1.0)	15 (1.2)
Communication				
Woman felt that health workers or staff did not listen and respond to her concerns*	71 (16.3)	100 (17.9)	41 (12.8)	212 (16.1)
Missing	5 (1.1)	3 (0.5)	8 (2.5)	16 (1.2)
Neglect and abandonment				
Staff member not present when the baby came out	14 (3.2)	2 (0.4)	9 (2.8)	25 (1.9)
Missing	0	2 (0.4)	2 (0.6)	4 (0.3)
Woman waited for long periods of time before attended by health workers*	178 (40.7)	66 (11.8)	43 (13.4)	287 (21.8)
Missing	1 (0.2)	1 (0.2)	0	2 (0.2)
Woman felt ignored, neglected, or that presence was a nuisance for health workers or staff*	64 (14.7)	62 (11.1)	65 (20.3)	191 (14.5)
Missing	0	3 (0.5)	0	4 (0.3)
Pain relief				
Requested pain relief*	61 (14.0)	183 (32.7)	34 (10.6)	278 (21.1)
Missing	7 (1.5)	0	1 (0.3)	8 (0.6)
Did not receive pain relief*	19 (31.2)	120 (65.6)	14 (41.2)	153 (55.0)
Missing	21 (34.4)	1 (0.6)	0	22 (7.9)

*$P < 0.05$

[1]Myanmar not included because 99.7% of women reported having a labour companion present

[2]Among women with vaginal birth only
